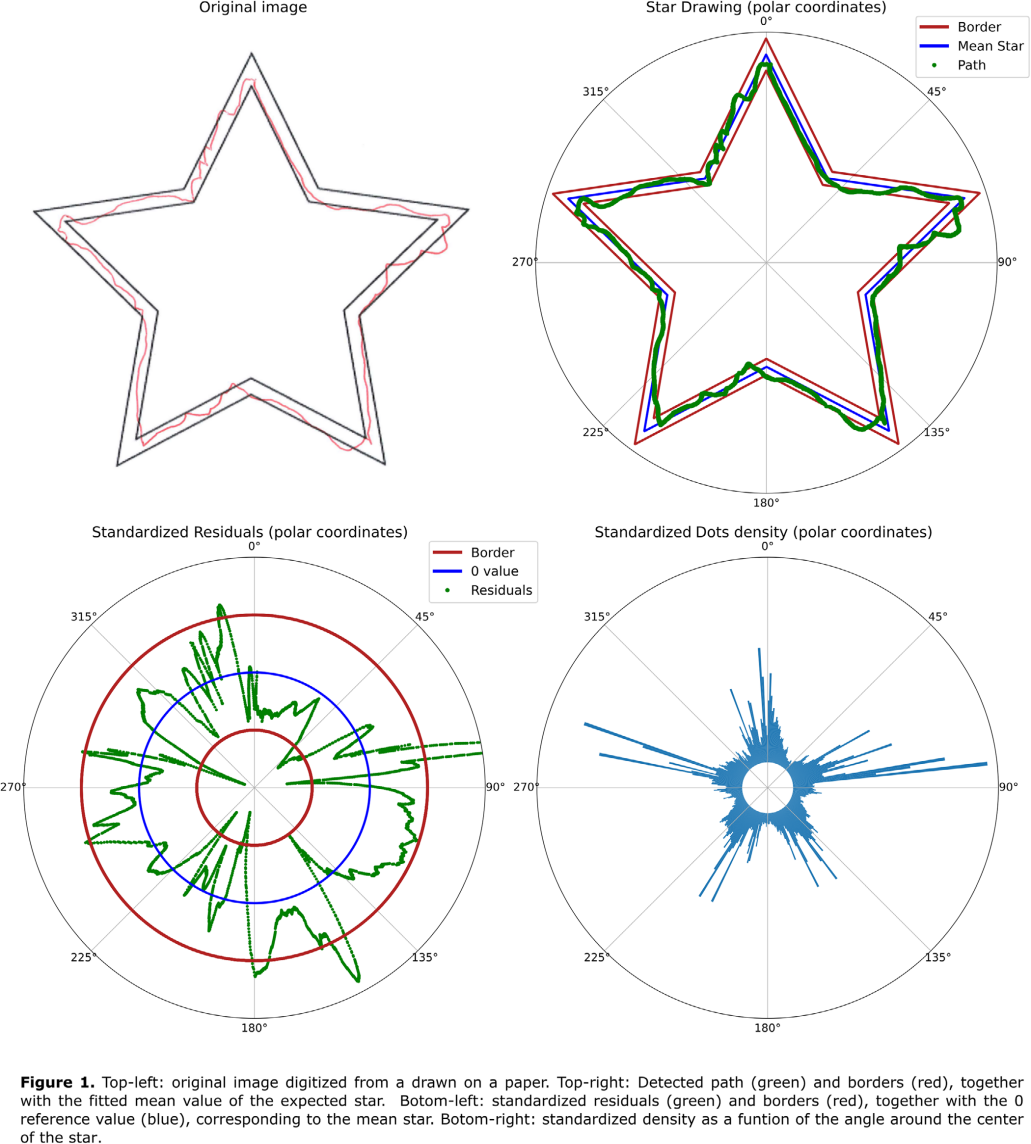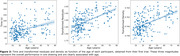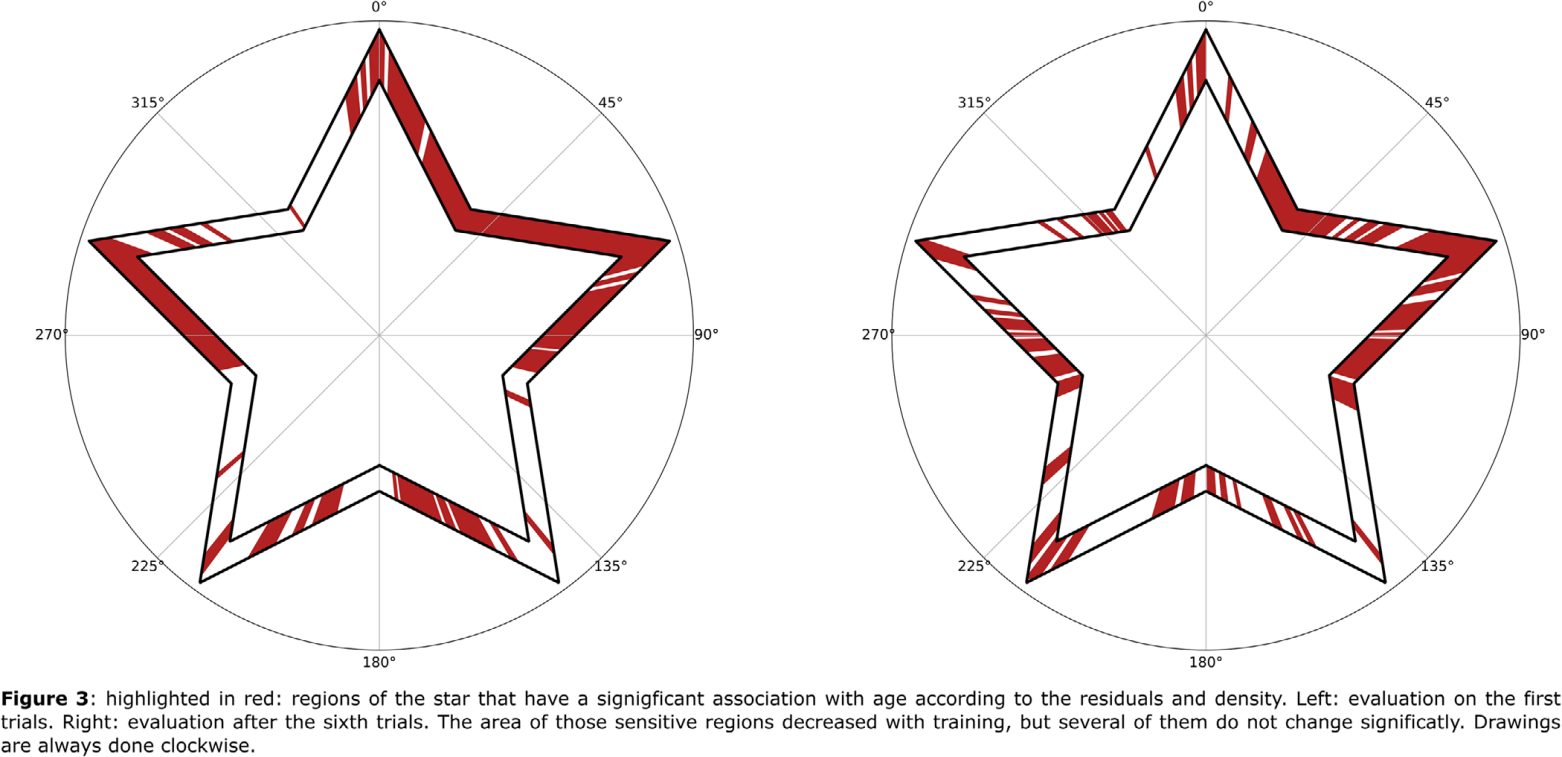# A novel analysis approach to Mirror Tracing Task performance to detect fine‐grained age‐related differences

**DOI:** 10.1002/alz.092273

**Published:** 2025-01-03

**Authors:** Pablo F. Garrido, Anne Cecilie Sjøli Bråthen, Emilie Sogn Falch, Jonas Kransberg, Øystein Sørensen, Kristine B Walhovd

**Affiliations:** ^1^ LCBC, University of Oslo, Oslo Norway

## Abstract

**Background:**

The Mirror Tracing Task (MTT) is a widely known test of motor learning. Participants are required to trace a star looking at their hand as a reflection in a mirror. In this work we introduce a new way of analysis beyond its simplicity.

**Method:**

Three versions of the MTT were employed in this project: paper‐based, online version, and an app version developed in the group, with over 3000 drawn stars and 170 participants. A generalization and standardization of the data is proposed to analyze all the possible stars and regular shapes. The proposed analysis method determines the distance between the drawn path and the mean star (the residuals), and the number of pixels drawn in a certain area (the density). These variables are measured for each unit of angle around the center of the star, providing us richer information about an MTT performance (Figure 1).

**Result:**

Residuals and density were summarized as single values representing the overall MTT performance. The sum of the density for all the angles and the sum of the absolute value of the residuals are used, respectively. They both correlate with the number of times a person draws outside the border, becoming a good alternative to it providing, at the same time, more complex information. Together with task time, these three variables show a clear positive association with age (all p‐values<0.001, see Figure 2). Through the study of the residuals and density as a function of the angle, areas of greatest difficulty for the participants can be identified. Moreover, a more refined analysis indicates that the results obtained in certain areas show a dependence on age (Figure 3).

**Conclusion:**

The proposed analysis provides an innovative approach to a simple task like the MTT. With this method, areas of special difficulty appear with age. These measures could be applied to the study of cognitive decline in aging or dementia, allowing us to look at the evolution of the performance with age and through the different trials.